# Hopping rim to rim through the Golgi

**DOI:** 10.7554/eLife.00903

**Published:** 2013-06-11

**Authors:** Suzanne R Pfeffer

**Affiliations:** 1 **Suzanne R Pfeffer**, an *eLife* reviewing editor, is at Stanford University, Stanford, United Statespfeffer@stanford.edu

**Keywords:** Golgi, Traffic, Membrane, Cell biology, Human

## Abstract

A novel approach based on tracking the fate of proteins that become ‘stapled’ to the walls of the Golgi yields insights into the long-sought mechanism of transport through this organelle.

**Related research article** Lavieu G, Zheng H, Rothman JE. 2013. Stapled Golgi cisternae remain in place as cargo passes through the stack. *eLife* **2**:e00558. doi: 10.7554/eLife.00558

**Image** Electron micrograph showing protein ‘staples’ (indicated by white arrows) distributed across the Golgi

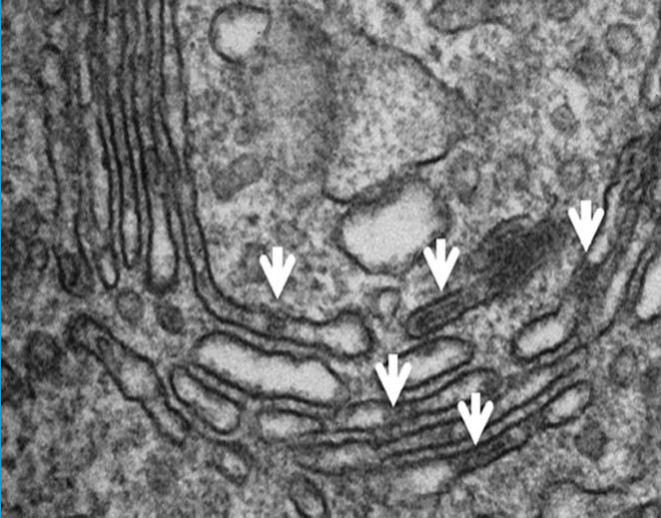

An unsolved mystery in biology is how proteins pass through the Golgi complex. The Golgi is comprised of a stack of 4–6 membrane-bound cisternae that house distinct sets of enzymes and lipids. Following synthesis in the endoplasmic reticulum (ER), most secreted and membrane proteins pass through the Golgi en route to their final destinations, and undergo modification by Golgi enzymes as they do so. There are currently two primary (and conflicting) models for how proteins traverse the Golgi (Emr et al., 2009). In one model, Golgi enzymes are stable residents of each cisterna and cargo moves from one compartment to the next. In the alternative ‘maturation’ model, the cargo is static and Golgi enzymes move between compartments. It has been difficult to distinguish between these models because it has not been possible to monitor the movements of both cargoes and enzymes concomitantly, in real time, in living cells. Now, in *eLife*, Gregory Lavieu, Hong Zheng and James Rothman, all at Yale University, use a novel technique to try to determine the mechanism of protein transport through the Golgi complex (Lavieu et al., 2013).

The approach used by Lavieu et al. is based upon the following logic. If a cargo can be trapped at a particular location in the Golgi, the maturation model predicts that the compartment housing that cargo will progress forward across the stack (Figure 1A). Lavieu et al. therefore created a chimeric membrane protein that could be delivered to different compartments of the secretory pathway in either a monomeric form that is efficiently transported, or an aggregated form that becomes trapped. The protein contained a domain that is designed to self-aggregate, but which can be triggered to disaggregate in the presence of a small molecule (Rollins et al., 2000).

**Figure 1.**
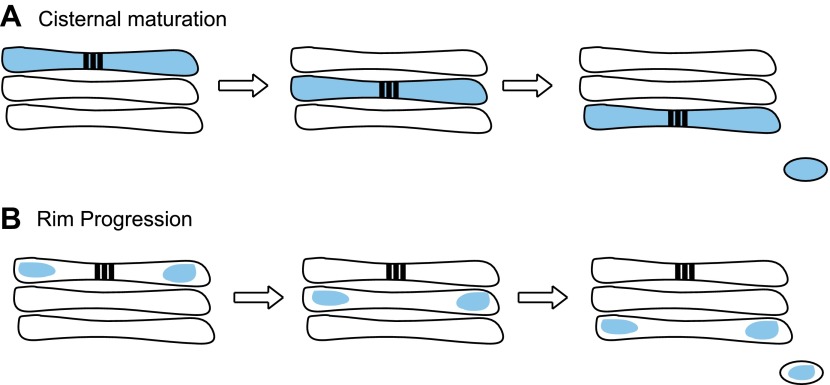
Two models have been proposed to explain transport through the Golgi. (**A**) In cisternal maturation, cargo (blue) moves across the stack of cisternae (three shown in each Golgi) and Golgi enzymes (not shown) move between the compartments to process the cargo before it exits the stack. (**B**) Lavieu et al. propose a model called rim progression, in which the edges of the cisternae (known as rims) move across the stack, while the central regions of the cisternae remain in place. They base this model on the observation that large protein aggregates called ‘staples’ (black bars in **A** and **B**)—which span the cisternae—remain stationary in the central regions, while soluble cargo (blue) progress across the stack via the rims.

When Lavieu et al. expressed the chimeric protein in its aggregated form in the ER, they found that this led to physical constriction of the organelle’s tubules—suggesting that the aggregates spanned the tubules. The electron-dense appearance of the aggregates (and their shape) led Lavieu et al. to name them ‘staples’. Importantly, the staples did not interfere with the transport of other proteins out of the ER.

By adding the disaggregating drug and manipulating the temperature, Lavieu et al. were able to permit transport of the protein out of the ER, just as far as the early or the late Golgi. Once the protein reached the desired location, they washed out the drug: this triggered aggregation, thereby trapping the protein. Immunofluorescence and electron microscopy, and glycosylation analysis, enabled Lavieu et al. to compare the fate of proteins that had been aggregated in precisely defined locations. They found that cargoes remained where they had been stapled, but without stapling, they moved to the cell surface.

The fact that the staples remain where they are generated strongly supports a model in which Golgi compartments are static, and cargo proteins move from one cisterna to the next during transport across the Golgi stack (Figure 1B). Nevertheless, there are important caveats. First, this experiment uses aggregates of an artificial protein, which ‘staples’ membrane compartments together. No natural cargo has ever been shown to behave in this way. Such aggregates could potentially inactivate entire subdomains of the Golgi itself. Lavieu et al. tried hard to control for this. They showed that Golgi complexes containing red fluorescent staples could still support the transport of a well-characterized cargo (GFP-labeled vesicular stomatitis virus G glycoprotein) to the cell surface. Cells harbouring stapled Golgi membranes were also able to deliver a larger cargo, collagen, from the Golgi to the cell surface, and to transport proteins to the ER upon addition of the fungal metabolite, brefeldin A. While these are excellent controls, the cells seem to dislike harbouring the chimeric proteins because they turn over in cells with a half life of about 6 hours (Lavieu et al., 2013). What cannot be determined with certainty is whether the control cargoes bypass a stapled microdomain.

Finally, the Yale team generated large aggregates of a cargo that did not become attached to the membrane, and showed that these aggregates moved through the membrane-stapled Golgi complex via the edges (or ‘rims’) of the cisternae. By contrast, the stapled membrane-associated proteins remained confined to the central regions of the Golgi (Figure 1B). Lavieu et al. conclude that rims progress forward from one cisterna to the next while the central regions of cisternae remain in place. This is reminiscent of an earlier study (Volchuk et al., 2000) that identified what appear to be ‘megavesicles’ carrying cargo from one region of the Golgi to another. Previous work from many labs has shown that a number of cargoes are found predominantly at the rims while Golgi enzymes seem to localize to the central domains of cisternae (see Cosson et al., 2002). Lavieu et al. suggest that others may have concluded that the Golgi matures because they were monitoring markers present at the rims; such analysis would have missed the fate of the central Golgi region. Lavieu et al. are careful not to over-interpret their findings but one conclusion seems quite clear: Golgi rims are distinct from static central regions, with broad implications for all models of Golgi transport.

Future work should focus on the mechanisms by which the cargo within Golgi rims moves forward and how it is segregated to the rim region. Rab family GTPases will surely be important (Mizuno-Yamasaki et al., 2012), and a cascade linking their functions may help contribute to the directionality of transport through the Golgi (Rivera-Molina and Novick, 2009; Pfeffer, 2010). Golgi rim transport also takes certain cargo backwards in a pathway to the ER. Thus, Golgi rims will need to accommodate the molecular machineries needed to separately package anterograde and retrograde cargoes. How Golgi residents are correctly localized and retained in the face of a large volume of secretory traffic remains an important question for future research.
